# Disproportionate, differential and targeted treatment: people who use drugs’ experiences of policing during the COVID-19 pandemic

**DOI:** 10.1186/s40352-024-00314-4

**Published:** 2025-01-23

**Authors:** Shelley Walker, Kasun Rathnayake, Paul Dietze, Peter Higgs, Bernadette Ward, Margaret Hellard, Joseph Doyle, Mark Stoove, Lisa Maher

**Affiliations:** 1 Burnet Institute, Melbourne, Australia; 2 National Drug Research Institute, Curtin University, Perth, Australia; 3 Monash University, Melbourne, Australia; 4 La Trobe University, Melbourne, Australia; 5 University of Melbourne, Melbourne, Australia; 6 The Kirby Institute, Sydney, Australia

**Keywords:** Drug use, COVID-19 pandemic, Policing, COVID fines, Penalty notices, People who use drugs, Criminalization

## Abstract

**Background:**

During the COVID-19 pandemic, governments worldwide introduced law enforcement measures to deter and punish breaches of emergency public health orders. For example, in Victoria, Australia, discretionary fines of A$1,652 were issued for breaching stay-at-home orders, and A$4,957 fines for ‘unlawful gatherings’; to date, approximately 30,000 fines remain outstanding or not paid in full. Studies globally have revealed how the expansion of policing powers produced significant collateral damage for marginalized populations, including people from low-income neighboorhoods, Indigenous Peoples, sex workers, and people from culturally diverse backgrounds. For people who use drugs, interactions with police are commonplace due to the criminalization of drug use, however, little empirical attention has been given to their experiences of pandemic policing; we aimed to address this gap in the literature.

**Methods:**

We conducted a mixed methods study involving participants of two prospective observational cohort studies of people who use drugs (*n* = 2,156) in Victoria, Australia, to understand impacts of the pandemic on their lives. Between 2020 and 2022 pandemic-related questions were added to survey instruments; during this period, 1,130 participants completed surveys. We descriptively analyzed the data of participants who reported police interactions related to pandemic restrictions (*n* = 125) and conducted qualitative interviews with 89 participants. Qualitative data were analysed thematically and integrated with quantitative results.

**Results:**

11% (*n* = 125) of survey respondents reported pandemic-related interactions with police; most (26%) were for breaching curfews and 30% received COVID-19 fines. Qualitative interviewees observed increased policing in street-based drug markets and local neighborhoods; many felt harassed and believed policing practices were targeted and discriminatory. Thirty-eight interviewees received COVID-19 fines; some were fined while homeless or travelling to or from harm reduction services. All lacked the financial means to pay fines, resulting in fear of additional criminalizing effects such as additional financial penalties, court orders, criminal convictions, and incarceration; for some fears became a reality.

**Conclusion:**

Our study demonstrates how public health emergency responses centred around policing and criminalization exacerbated harms for people who use drugs, with detrimental effects enduring beyond the pandemic. Results provide insights for future public health emergencies, including highlighting the need for responses that protect, rather than abrogate the health and safety needs of marginalized and criminalized groups.

## Background

In response to the COVID-19 pandemic (herein referred to as the ‘pandemic’) governments worldwide introduced law enforcement measures, including penalty notices (commonly known as ‘on-the-spot’ fines), to deter and punish breaches of public health orders. The expansion of policing powers during the pandemic produced various direct and indirect adverse impacts with significant collateral damage for vulnerable and disadvantaged populations (Brooks & Lorange, 2023; Fazio et al., 2022; Iversen et al., 2020; Russell et al., 2022; Skolnik, 2020).

Pandemic policing created opportunities for the expansion of longstanding and selective criminalizing processes and the intensification of socio-economic and racialized inequities for key populations (Boon-Kuo et al., 2021a; Kajeepeta et al., 2022; Williams et al., 2021). Culturally and ethnically diverse groups, Indigenous peoples, sex workers, and people residing in low socio-economic neighborhoods and/or public housing, were among the populations more likely to be impacted (Boon-Kuo et al., 2021; Emmer et al.; Fatsis & Lamb, 2021; Harris et al., 2022, 2023; Kajeepeta et al., 2022; Lelliott et al., 2021). Members of these populations were more likely to be stopped by and subjected to police violence, falsely accused of pandemic rule-breaking and issued with associated penalty notices (Boon-Kuo et al., 2021b; Harris et al., 2023; Hopkins & Popovic, 2023; Kajeepeta et al., 2022; Leal et al., 2023; Parliament of Victoria, 2021).

For example, US studies found penalty notices related to physical distancing breaches were more likely to be issued to Black and Latina people (Kajeepeta et al., 2022; Leal et al., 2023), and Black and Hispanic people were also more likely to be stopped and frisked (Khan et al., 2021). In 2020, Black people in London were reportedly 11 times more likely to be stopped by police than white people (Williams et al., 2021), and a qualitative UK study found that people from racially marginalized groups and low-income communities experienced unevenly distributed police harassment, intimidation, provocation, and violence during the pandemic (Harris et al., 2023). Similarly in New South Wales (NSW) Australia, 74% of all First Nations people who were stopped by police for pandemic-related incidents were searched compared to only 45% of non-Indigenous peoples (Boon-Kuo et al., 2021). Furthermore, in the state of Victoria, crime statistics revealed Sudanese and South Sudanese born people were 35.6 times more likely to be issued with pandemic-related fines (herein referred to as COVID-19 fines) than other Victorians and First Nations people were 4.5 times more likely to be issued with a fine than non-Indigenous people (Hopkins & Popovic, 2023). Global studies have highlighted how sex workers were particularly vulnerable to police arrest and mistreatment during the pandemic (Benoit & Unsworth, 2022; Platt et al., 2020), including being subject to punitive crackdowns, raids on homes, and threatened deportation (Fowler et al., 2023). Moreover, as streets and public spaces are frequent venues of sex work, their visibility meant the odds of interactions with police were increased (Aristegui et al., 2022; Bromfield et al., 2021; Iversen et al., 2020).

For people who use drugs, interactions with law enforcement are commonplace due to the criminalization of drug use (Hughes et al., 2018; Maher & Dixon, 2017; Mostyn et al., 2012), resulting in a plethora of adverse impacts on their mental, physical, psychological and social health (Chiu & Burris, 2012). Drug use criminalization increases stigmatizing attitudes and discrimination towards people who use drugs, which exacerbates social exclusion and poor mental health (Chandler et al., 2009; Maher & Dixon, 2017). These stigmatizing effects also prevent people who use drugs’ accessing drug treatment and harm reduction interventions—such as opioid agonist treatment [OAT], needle and syringe programs (NSPs) and HIV antiretroviral therapy—which increases their risk of exposure to drug-related harms (DeBeck et al., 2017; Wolfe et al., 2010). Furthermore, criminalization of drug use artificially inflates the costs of illegal drugs, which means people who use drugs sometimes resort to crime or potentially risky activities such as sex work (which is often criminalized) to support their drug use (Maher et al., 2002), which in turn increases their risk of arrest and imprisonment (Dolan et al., 2016). Because the street-based drug market areas and physical spaces that people who use drugs occupy to procure and use drugs are often publicly visible, interactions with police are also increased, which exacerbates health and social risks (England, 2008; Kurcevič, 2023). Despite that people who use drugs are more likely to experience interactions with police, to our knowledge no previous studies have specifically examined the effects of pandemic-related policing on their lives.

Similar to other disadvantaged and marginalized populations during the pandemic, people who use drugs were considered more vulnerable to a range of adverse impacts (Dietze & Peacock, 2020; Dunlop et al., 2020; Iversen et al., 2021; Price et al., 2021; Rathnayake et al., 2023; Walker et al., 2023). Residing in over-crowded crisis housing or being homeless made physical distancing and social-isolation difficult, and thus increased the risk of SARS-CoV-2 exposure (Scallan et al., 2022; Varshney et al., 2022). It was also perceived that fear of discrimination and stigma from healthcare professionals exacerbated poor access to healthcare for those who experienced COVID-19 symptoms (Dietze & Peacock, 2020). Furthermore, social distancing requirements and travel restrictions further reduced people who use drugs’ access to harm reduction and drug treatment services, which increased their risk of exposure to opioid overdose, injecting-related injuries and disease, and blood-borne viruses (Croxford et al., 2021; Efunnuga et al., 2022). Our previous work also demonstrated how the pandemic produced unintended adverse socio-economic consequences for people who use drugs (ref removed for review process), including increased financial hardship and withdrawal stress due to drug market shifts that resulted in increased drug prices (ref removed for review process).

The aim of this study was to extend understandings of the impacts of pandemic policing on the lives of people who use drugs, with a focus on their experiences of interactions with police and receiving COVID-19 penalty notices. In doing so, we widen the evidence of the multiplicity of adverse implications of the introduction of law enforcement measures to compel compliance with public health orders during a global pandemic, for a population that has received scant attention in the literature.

Below, we outline the methods used for our study, including the research setting and how data were collected and analyzed, followed by research findings and a discussion of their implications.

## Methods

We used a mixed methods research design (Fetters et al., 2013; Guetterman et al., 2015), which involved collecting and analyzing quantitative survey responses and qualitative interview data from participants of two long running community-based prospective observational studies of people who use drugs.

### Research setting

This study is based in the state of Victoria, the second smallest and second most-populated state in Australia. The city of Melbourne, in Victoria, where approximately three quarters of study participants lived during the study, experienced what has been characterized as one of the world’s longest cumulative ‘lockdowns’ (McLaren et al., 2023). Melbourne had six lockdown periods between March 2020 and October 2021, involving more than 260 days of high-level containment measures, including ‘stay at home orders’ that involved only being allowed to leave home for essential purposes (i.e., care/caregiving, exercise for limited times, authorized work/study, medical appointments, shopping for essential items) (McLaren et al., 2023). Night-time curfews and travel restrictions were differentially enforced, including being unable to travel more than five kilometres from home. In rural and regional Victoria, lockdown periods were fewer, and restrictions less stringent than for Melbourne.

With the aim of enforcing public health orders, Victorian police were given discretionary powers to issue ‘on-the-spot’ COVID-19 fines of A$200 for failing to wear a facemask in public spaces, A$1,652 for breaching stay-at-home orders and A$4,957 for ‘unlawful gatherings’; fines could be extended to A$10,000 for repeat offenders (Elkins, 2022) Between 2020 and 2021, more than 50,000 COVID-19 fines were issued in Victoria, totalling more than A$10 million (Taylor, 2022). Following concerns by community organisations, public commentators and human rights organisations, that many individuals lacked the financial means to pay fines, and that some had been issued to people acting in accordance with the law (Boon-Kuo et al., 2021a; Lelliott et al., 2021; McGowan, 2022) some Australian states withdrew a portion of fines. For example, in 2022 the NSW Government withdrew approximately half of the 62,000 COVID-19 fines issued, and in 2024 they withdrew the remaining fines (NSW Government, 2024); and in 2023 the Victorian Government withdrew less than a quarter (11,800) of the 50,000 fines issued (Public Accounts and Estimates Committee, 2023). In Victoria, processes are available for challenging the validity of COVID-19 fines in court (Mabin, 2023), however, in June 2023, approximately 30,000 fines that were issued, were outstanding or had not been paid in full (Public Accounts and Estimates Committee, 2023) and to our knowledge, the situation has not changed since that time.

### Quantitative studies

Quantitative data were sourced from the Melbourne Injecting Drug User Cohort Study (SuperMIX) (Van Den Boom et al., 2022), and the Understanding Methamphetamine Use in Victoria Study (VMAX) (Quinn et al., 2021). SuperMIX (*n* = 1,303, at time of analysis) was established in 2008 and is focused on the health and socio-economic outcomes and drug use patterns of people who inject drugs (Van Den Boom et al., 2022). Study eligibility includes being over 18 years of age at recruitment, injecting at least monthly for six months prior to baseline interview and residing in Melbourne. VMAX (*n* = 853, at time of analysis) commenced in 2016 and examines long-term patterns of methamphetamine use and the effects on service use and health and well-being outcomes (Quinn et al., 2021). Eligibility includes being at least 18 years of age; residing in Melbourne or rural/regional Victoria; and using methamphetamine predominantly via non-injecting routes of administration (e.g., smoking, snorting) at least monthly in the last six months. Participants of both studies complete annual follow-up surveys via phone call or in-person and are paid A$50 for each survey completed to cover time and out-of-pocket expenses.

Pandemic-related questions were added to the SuperMIX and VMAX surveys from March 2020 to May 2022, with the aim of understanding the impact of the pandemic and associated restrictions on participants health and well-being (e.g., patterns of drug use, health outcomes, health service use, and interactions with law enforcement). Questions pertaining to interactions with law enforcement included: ‘Have you had any recent interactions with police related to COVID-19 restrictions?’ (Yes/No). ‘If yes, what did this restriction relate to?’ (‘breaking rules of gatherings in homes’, ‘breaking rules on gatherings in public’, ‘failing to wear a face mask’, ‘breaking curfew’, or ‘other/don’t know/not applicable’), and ‘what was the outcome of the interaction?’ (‘issued with a warning’, ‘issued with a fine’, ‘simple discussion’, or ‘arrest’). During this period, 1,130 SuperMIX and VMAX participants completed at least one follow-up survey. Of this sample, cross-sectional descriptive statistics were generated for those who reported at least one interaction with the police in relation to pandemic restrictions.

### Qualitative studies

Between August 2021 to April 2022, the first author (SW) conducted in-depth interviews with 38 SuperMIX and 38 VMAX participants who completed surveys during the pandemic. We used an ethno-epidemiological (‘ethno-epi’) random sampling recruitment technique with the aim of increasing the generalizability of the results across the cohort samples (Walker et al., 2024). Investigation topics included impacts of the pandemic and associated restrictions on experiences of housing, employment, and income status; social relationships and support; access to health services; drug use; and interactions with law enforcement - the latter of which are the focus of this article. In-depth interviews were conducted via mobile phone or video-call (*n* = 71) and in person (*n* = 5) and lasted an average of 40 min. Interviewees received A$40 cash for their time and out-of-pocket expenses. Interviews were audio-recorded and transcribed verbatim.

In January 2024, additional brief interviews (*n* = 13) were conducted by the first author (SW) via telephone call, to understand any ongoing impacts of receiving COVID-19 fines. Our eligible sample were SuperMIX and VMAX participants who reported receiving at least one COVID-19 fine during the study period (*n* = 38). Attempts were made to contact all eligible participants via phone call; ten SuperMIX and three VMAX participants completed an interview. Interviews involved five brief questions: (1) how many fines were received; (2) what restrictions fine/s were related to; (3) what the dollar value of fine/s was; (4) had fine/s been paid; and if not (5) what they were planning to do about pending fine/s if anything. Interviews lasted an average of 10 min, and interviewees received A$20 for their participation. Detailed notes were transcribed for each interview.

The qualitative data analysis was led by SW. Interview transcripts (*n* = 89) were thematically analyzed using Neale’s Iterative Categorisation technique; a rigorous, flexible non-linear systematic process developed within the field of addiction (Neale, 2016, 2021). NVivo qualitative software (QSR International, 2020) was used to organize data. Deductive codes were created that reflected study aims and available literature about pandemic-related interactions with police. Data extracts were assigned to related codes and as additional themes were identified, new codes and sub-codes were established to represent these, producing a hierarchical ‘tree of codes’ (Neale, 2016). A process of differentiating extracts was used to check for similarities and differences within individual responses, and between participant accounts. Once this process was complete, coded data were exported into Word documents (one for each overarching high-level code) and extracts of data were summarized following a systematic inductive line-by-line process, which also involved identifying any duplication, complementarity, and contradiction between codes. We actively monitored the literature for newly published studies to ensure our findings were informed by recent established knowledge, and new ideas were formulated iteratively as findings were written up (Neale, 2016, 2021). Themes were iteratively refined based on ongoing feedback and review from the core research team (LM, PH and PD). Final themes were agreed upon by all authors. All identifiable information has been removed from direct quotes. Quotations are followed by a pseudonym, participant geographical location (metro or regional), and a unique identifier.

### Quantitative and qualitative data integration

Quantitative and qualitative data were collected and analyzed separately in parallel to each other, followed by an integrated analysis (Fetters et al., 2013). The process of integration involved comparing the data to determine how they diverged or complemented each other, with the aim of drawing insights and extending understandings beyond what could be achieved via the separate analysis of the two data sources (Guetterman et al., 2015). We use a mixed methods ‘joint display’—a methodological approach for integrating or merging mixed methods findings (Guetterman et al., 2015; Fetters, 2022)—which we present via a table to highlight the summarised key differences and similarities between the quantitative and qualitative data sets.

## Results

Below, we present quantitative results, followed by qualitative findings, and a ‘joint display’ (Guetterman et al., 2015), of the key differences and similarities between the two data sets.

### Quantitative results

11% of participants who completed SuperMIX or VMAX follow-up surveys during the study period (125/1,130), reported at least one interaction with police in relation to pandemic restrictions, including 9% (*n* = 40/430) of women and 12% of men (85/700). Most participants (80%) who reported interactions with police were living in Melbourne. Nineteen participants who reported interactions with police identified as Aboriginal and Torres Strait Islander (15%), and most (82%) had ever injected drugs (90% women vs. 78% men). Experiences of significant socio-economic disadvantage were common among the sample. At the time, most participants who completed surveys during the study period experienced socio-economic disadvantage, however, levels of disadvantage were slightly higher for those who reported interactions with police compared to those who did not. At the time of their most recent interview, more than a quarter (26%) were living in unstable housing, including some who were homeless. Most participants (79%) were unemployed, and more than half (58%) reported a history of incarceration (65% men vs. 40% women). The percentage of participants who reported interactions with police who had ever overdosed (37%) was more than twice the percentage of those who did not (15%), and this was higher for men (29%) than women (13%). See Table 1.

**Table 1:** Survey participant characteristics

Participant characteristics	Reported interaction with police			Did not report interactions with police		
	Women (*n* = 40) *n* (%)	Men (*n* = 85) *n* (%)	Total (*n* = 125) *n* (%)	Women (*n* = 383) *n* (%)	Men (*n* = 622) *n* (%)	Total (*n* = 1005) *n* (%)
Aboriginal and Torres Strait Islander	6 (15)	13 (15)	19 (15)	46 (12)	88 (14)	134 (13)
Non-Australian born	6 (15)	11 (13)	17 (14)	29 (8)	91 (15)	120 (12)
Living in Melbourne	32 (80)	68 (80)	100 (80)	283 (74)	490 (79)	773 (77)
Living in rural/regional Victoria	8 (20)	17 (20)	25 (20)	100 (26)	132 (21)	232 (23)
Completed > year 10	29 (72.5)	52 (61)	81 (65)	259 (68)	414 (66.5)	673 (67)
Ever incarcerated	16 (40)	56 (66)	72 (58)	162 (42)	392 (63)	554 (55)
Unemployed	31 (77.5)	66 (80)	99 (79)	282 (74)	503 (81)	785 (78)
Unstable housing	8 (20)	25 (29)	33 (26)	65 (17)	171 (27.5)	236 (23.5)
Prescribed OAT	17 (42.5)	31 (36.5)	48 (38)	139 (36)	210 (34)	349 (35)
Ever overdosed	9 (22.5)	25 (29)	34 (37)	65 (17)	82 (13)	147 (15)
Ever injected drugs	36 (90)	66 (78)	102 (82)	310 (81)	541 (87)	851 (85)

The most common reasons for interactions with police were related to breaking curfews (26%), failing to wear a facemask (16%), and breaching travel restrictions (13%). Women were much more likely than men to have interactions with police related to ‘gatherings in homes’ (22.5% vs. 3.5%), and ‘failing to wear a facemask’ (22.5% vs. 14%). For those who reported interactions with police, 30% received a COVID-19 fine and 15% reported being arrested; ‘simple discussions’ were the most common outcome (41%), which was more likely for women (57%) than men (34%). See Table 2.

**Table 2:** Survey responses to pandemic-related questions

Survey responses	Women	Men	Total
1. At least one police interaction related to pandemic restrictions	*n* = 40 (%)	*n* = 85 (%)	*n* = 125 (%)
2. Restriction the interaction related to			
• Gatherings in homes	9 (22.5)	3 (3.5)	12 (10)
• Gatherings in public places	3 (7.5)	8 (9.4)	11 (9)
• Failing to wear a mask	9 (22.5)	12 (14)	21 (16)
• Breaking curfew	8 (20)	25 (29)	33 (26)
• Breaching 5 km radius*	4 (10)	12 (14)	16 (13)
• Leaving home for non-essential purpose*	2 (5)	7 (8.2)	9 (7)
• Other	6 (15)	11 (13)	17 (21)
3. Outcome of interaction			
• Simple discussion	23 (57.5)	29 (34)	51 (41)
• Issued with a warning	10 (25)	18 (21)	28 (22)
• Issued with a fine	12 (30)	30 (35)	38 (30)
• Arrest	7 (17.5)	12 (14)	19 (15)

*Free text survey responses

### Qualitative results

More than a third of in-depth interviewees (28/76) reported interactions with police in relation to the pandemic, including 14 men and 14 women; their median age was 34 years. Participants who reported interactions with police were approximately twice as likely to identify as Aboriginal and Torres Strait Islander (11% vs. 6%) and non-Australian born (7% vs. 4%) than those who did not; they were also one third more likely to have ever been incarcerated (68% vs. 42%); and twice as likely to be living in unstable accommodation (36% vs. 17%). Furthermore, in-depth interviewees who reported interactions with police were twice as likely to have ever over-dosed (25% vs. 12.5%) (Table 3)

**Table 3:** In-depth interview participant characteristics

Participant characteristics	Interactions with police (*n* = 28) *n* (%)	No interactions with police (*n* = 48) *n* (%)
Aboriginal and Torres Strait Islander	3 (11)	3 (6)
Non-Australian born	2 (7)	2 (4)
Living in Melbourne	22 (79)	34 (71)
Living in rural/regional Victoria	6 (21)	14 (29)
Completed > year 10	20 (71)	32 (67)
Ever incarcerated	19 (68)	20 (42)
Unemployed	24 (86)	32 (67)
Unstable housing	10 (36)	8 (17)
Prescribed OAT	15 (54)	50 (50)
Ever overdosed	7 (25)	6 (12.5)
Ever injected drugs	24 (86)	38 (79)

Of the 13 participants who completed brief qualitative interviews in relation to receiving COVID-19 fines, nine were men and four were women. Most were living in Melbourne (*n* = 12). Nine participants were living in unstable housing, and all but one was unemployed (*n* = 12).

Qualitative data from in-depth interviews (*n* = 76) and brief interviews (*n* = 13) are presented via the following themes: (1) increased police presence in the “hoods”; (2) Healthcare rights denied; (3) “Yeah … we’re targeted!”; and (4) COVID-19 fines, arrest, and prison.

### Increased police presence in the “hoods”

Several participants observed an increased police presence in the suburbs where they lived and areas where they purchased and used drugs. A constant fear that they would be unfairly “targeted” or “picked up by the police” because they were known to use drugs and had criminal legal histories, was a common narrative.

Jen, who received a fine of A$200 for not wearing a facemask and another fine for stealing chocolate during the pandemic said:

*During COVID it was full on*, *with coppers everywhere. Personally*, *I think it was a quota thing*, *like they’re trying to get their numbers up*, *especially in the high rises and certain spots in the street. But I think once you’ve had a bit of a hard time from the coppers*, *you always do worry that you’ll be more singled out. But yeah*, *the police*, *the presence in this area was through the roof. Heaps of people were getting arrested*, *but just for the littlest things. Like for example I got pulled over for shoplifting … like it was for chocolate*, *and made a really big thing of*, *and I got a fine. In the past that wouldn’t have happened. But if they know you’ve done time and you’re in the drug scene you’re targeted*, *but especially through COVID. Like I said*, *the police presence*, *it was like … we were more watched. (Jen*, *metro*, *#24)*

Tara, who was dependent on heroin and lived in public housing in a low socio-economic area, said she felt like police used their additional powers during the pandemic as an “excuse to hassle” people in the poorer suburbs like the one where she lived:

*I feel like in places like this*, *all the people in the hoods*, *they’re fighting for survival in those streets you walk around. When COVID came it was … obviously [the police] want to come to the hoods and police the people … but then I don’t think it was like the fear of the COVID … it was almost like it’s an opportunity to deal with all the so called ‘scum of the earth’*, *like just finding excuses to hassle them. (Tara*, *metro*, *#37)*

Although several participants who completed in-depth interviews described “sticking to the rules”, breaching pandemic-related public health orders to purchase drugs was an activity many were forced to do. This usually involved travelling further from home than allowed during lockdown periods or leaving home for non-essential purposes. While most avoided police contact when procuring or purchasing drugs, almost all said they lived with the constant fear that they would be “pulled over” by police or fined, especially in street-based drug market areas where the police presence was considered greater.

*Sometimes I would have to obviously go out of the five k’s (km’s) to get weed or whatever. Stressful*, *you know […] because you’ve got the issue of getting pulled over at any time and they were always hanging around the suburbs where I go. Yeah*, *every time I see a cop car*, *you know*, *you just worry. But look*, *the reality is people have dependencies on illicit drugs … if they’re not able to access those drugs there are consequences*, *health consequences*, *you know*, *mental health*, *physical health consequences. (Sergio*, *metro*, *#69)*

### Healthcare rights denied

Many participants were required to travel outside restricted travel zones and curfews to access drug treatment and harm reduction services during the pandemic. Some who were approached by police at these times described experiencing a lack of care and concern for their health and wellbeing.

Despite travelling with healthcare documentation (e.g., medical certificates, NSP cards) which meant they were exempt from pandemic travel restrictions, some continued to be stopped by police and fined. For Davey, the pharmacist where he collected his daily methadone was just outside the restricted travel zone; he was stopped by police several times while travelling there. He said, “Any time the police stopped me, it was like, ‘Why are you here?’” [and] I’d just show them my methadone [card] and go “Well, look, I’ve come to get this”, and usually they’d leave me alone”. However, one day he and his friend were stopped by police, searched, and fined:

*It was full on*, *just harassment really. Like for some reason they obviously thought I had [drugs] on me*, *because they searched me down to where they took my socks and shoes off in the street […] I said*, *“can we just go round the corner”*, *I said*, *like*, *“My mum’s friends drive past here. Like I’ve lived here for 41 years”. So he goes*, *“You got something to hide? That sounds suspicious”. Like … they could see on my records*, *that I’ve never … you know I’ve never been to jail. I’ve got speeding tickets*, *I’ve lost my licence*, *but yeah*, *no*, *it was shit. They didn’t find anything*, *and then fined me and my friend … it was like Section 199 of the blah-blah-blah … just jargon for some social distancing thing because we didn’t live together […] I had a mask on*, *yeah*, *and he did too*, *yeah. It was a $1800 fine […] and it was so obvious that they were looking for drugs of some sort*, *because first when I asked what it was about*, *they said “Oh*, *your mate was driving erratically” […] It’s a pity they didn’t find what apparently*, *they thought I had. It felt like they were doing everything they could to find something*, *but all they could get was social distancing. And have I paid it*, *no bloody way! (Davey*, *metro*, *#25)*

A commonly expressed narrative was also one of not feeling believed when approached and queried by police when travelling to or from harm reduction or drug treatment services. For example, Natasha who was homeless during the pandemic was approached by police on her journey to the NSP. She was asked to provide her address which she believed was within 5km of the NSP. The police not only disagreed, but said Natasha was being dishonest about visiting the NSP, and issued her with a fine:

*I said to [the police]*, *“How can you be outside if you sleep outside?” They said*, *“Well wherever your address is for Centrelink”—which was [the health service]—“you’re not allowed 5k out of that”*, *which I wasn’t anyway. So*, *I said*, *“Why am I copping a fine*, *because [the health] service”*, *I said*, *“that’s in the 5k’s”*, *and they said*, *“No it’s not*, *and we know you’re not down here for that anyway”. (Natasha*, *metro*, *#20)*

### “Yeah … we’re targeted!”

Given most participants had histories of criminal legal involvement, interactions with police prior to the pandemic had been commonplace, as was the feeling they had been unfairly targeted by police previously. During the pandemic, however, many participants described feeling like they were stopped by police and singled out for questioning or compliance checks more than usual. Several participants believed police had used their powers unreasonably, and that the pandemic had simply provided an “excuse” for police to target people who use drugs.

For example, Ben and his two friends were fined A$1,350 each for being outside their homes just after a midnight curfew. Ben believed the police could have used their discretionary powers in this instance; he was “dumbfounded” at the amount of the fine, and felt they were deliberately targeted:

*You couldn’t go out after 12 o’clock … a pandemic thing in lockdown. They’d stopped it*, *and then it was back in*, *but I didn’t know. It was half an hour past 12 o’clock. I was out with my mates. We got pulled over and they said*, *“Oh*, *the law’s come back in. You’ve got to be in for 12”. I was*, *like*, *“What?” I said*, *“It’s only half past 12*, *mate [and] we’re going home”. They charged the five of us $1*, *350 each. To be honest*, *I felt dumbfounded. Yeah*, *in a way I felt*, *like targeted. I felt like I was picked on. (Ben*, *metro*, *#60)*

Seven participants reported being searched by police when they were approached about breaching public health orders. Most said the treatment they received was “typical” and “expected”; an experience they were resigned to because they had been stopped and searched by police many times previously.

When confronted with what felt like discriminatory and unjustified behaviour by police, some participants said they knew how to behave passively to avoid aggravating the situation. Christopher said he had “been in trouble” with police many times since his teens. He was stopped and searched by police after visiting the NSP, and despite feeling like their treatment towards him was discriminatory, he said “I just pull right back”, to avoid giving them more reason to charge or arrest him:

*I got pulled over […] coming back from [the NSP]. Luckily*, *they didn’t know who I was cos I’d been out of trouble for a few years … but I knew their faces from back in the day. […] Instead of just asking what I was doing*, *like “You shouldn’t be out”*, *you know*, *they were just being arseholes in their questions and all that … but nothing out of the ordinary […] I had a heap of fits down my pants*, *and they did a pat down*, *but luckily*, *they didn’t find anything. […] I just pull right back and*, *“Yep*, *sir*, *no sir”. They were probably trying to get a rise out of me so they could do something*, *and it didn’t happen for them*, *so they left me alone. (Christopher*, *metro*, *#32)*

Darren, who had been using heroin all his adult life and had a history of incarceration, remembered being stopped and searched by police on the train at least twice during the pandemic. Once was after purchasing heroin in a suburb “just outside the restricted 5k travel zone” and the other was while travelling to collect sterile injecting equipment. He described the police as unnecessarily “aggressive”:

*They ask you for your license and do a pat down. Yeah*, *they were … full on*, *full on. I had gear on me too*, *but I had it fairly well hidden in a thing in my bag. But*, *yeah*, *they were very aggressive*, *but at least I knew my rights and all that […] The second time I had a NSP card thing saying that this person is entitled to travel for medical purposes. But yeah*, *both times were terrible […] But yeah … we’re targeted. Like I say*, *you get off at [that suburb] … sometimes there’s four police there. They’re just enforcing fines and inspecting tickets at the low socio-economic ending of trains. (Darren*, *metro*, *#3)*

### COVID-19 fines, arrests and prison

Of the 31 interviewees who received COVID-19 fines, at least five received more than one fine, including one participant who received at least four fines. At least six participants received at least one AUD$200 fine for failing to wear a facemask, and 12 received fines of A$1,695 for allegedly breaching lockdown restrictions such as being outside restricted travel zones, breaching curfews or leaving home for non-essential purposes.

All but three participants who received COVID-19 fines had not paid them; all said they lacked the financial means to do so. Brandon who received a fine of A$200 for failing to wear a facemask while pan-handling, and a A$2,800 fine for breaching travel restrictions, had not paid them. When asked why, he said, “Because I haven’t got any money”. He said:

*So*, *when the COVID first started*, *I had travelled to score*, *and the fine was something stupid*, *a couple of grand for being so much distance from my home. It was after the curfew*, *and it was $2*, *800. Yeah*, *and fines for no mask. I was scabbing money*, *at the time*, *yeah. (Brandon*, *metro*, *#21)*

Three participants who had not paid their fines were ordered to attend court. Mandy, a peer worker whose role involved supporting people who inject drugs to attend healthcare appointments and harm reduction services during the pandemic, received a fine for breaching travel restrictions while taking a client to their methadone provider. She said they were stopped by police at a regular road check, and “[the police] looked at [her] permit [and her] roster, but still issued a fine”. When asked why she thought they issued a fine despite being exempt, she said, “I don’t know, they’re just pricks. The police have never really helped me much. They see my record and stuff and then they just don’t care”. Mandy forgot about the fine and subsequently received a court order. At the time of interview, she was in the process of contesting the charge:

*Because of COVID and all that*, *the court case got adjourned and I lost track of it [and] I missed the court date*, *and now it’s on my police check*, *as basically not following police directions … even though I had my permit*, *my roster*, *and I was in my work clothes […] I did call Fines Victoria saying I wanted to contest them*, *[but] they said*, *“No”. I called the courts at one stage*, *but I couldn’t get through […] and then I applied for a police check*, *just for work purposes*, *and I couldn’t believe that it was on there. […] I’m going down that avenue now to see if I can find a way of contesting it*, *because I don’t need that on my police check or record. (Mandy*, *metro*, *#22)*

Only four participants said they were planning to or had applied to have their fines waived. Sarah, who had prior convictions related to her heroin use, was fined for breaching a curfew. Her fine was waived, but she was required to complete compulsory drug counselling which she said was “basically a waste of time” as a condition of the waiver.

Amongst participants who were planning to, or were in the process of contesting fines, all assumed their attempts would be futile; due mostly to previous unsuccessful experiences or of feeling unsupported when contesting legal processes. Lila had received numerous fines during lockdowns and was hoping to context them:

*Yeah*, *it’s been shit […] I had a letter from my case workers and the doctor saying that I’m on methadone and I have to go get my scripts and see the doctor. Yeah*, *bottom line*, *they didn’t accept it. They still sent me out a fine. So*, *I’ve got like four fines sitting here […] I’m gonna to do my best to not have to pay*, *but at the end of the day*, *they’re government authority and I’m a nobody. So*, *the chances of me winning would be slim to none. (Lila*, *metro*, *#56)*

Two participants reported that they had served prison time for pandemic-related breaches. Barbara was on a community corrections order when she was stopped by police at a road check while travelling to a pharmacy to get paracetamol for her sick child. She said she was arrested because she challenged the police’s decision. She was bailed to the community but missed her court appearance and was thus remanded to the women’s prison, where she spent one month. Dave received a concurrent prison sentence in lieu of his fine, while in prison for other offences.

### Integrated findings

Interview participants were four times more likely than survey respondents (44% vs. 11%) to report interactions with police, underscoring the importance of qualitative insights in capturing more nuanced, personal accounts that might not be fully expressed through survey responses alone. Quantitatively, breaches of curfews (26%) and failure to wear facemasks (17%) were frequently cited reasons for police interactions. The qualitative findings reinforce this but also provide context, highlighting how these interactions with police were perceived as targeted, especially in low socio-economic areas and drug market areas where participants lived and spent time.

Although data alignment is evident in outcomes of police interactions—30% of survey respondents and qualitative participants 30% of survey respondents and 35% of qualitative participants reported receiving fines—the qualitative data illustrate the financial and emotional toll on individuals who felt targeted and harassed and were unable to pay fines, and the frustration and helplessness experienced in attempting to contest fines. While quantitative data indicate that 15% of participants were arrested and 41% received only a warning or advice, qualitative findings shed light on the emotional and social consequences of these interactions and how pandemic-related charges led to further entanglement with the criminal legal system.

Overall, these findings underscore how quantitative results offer a broad view of pandemic-related police interactions, while qualitative data provide a more nuanced in-depth understanding of the personal and systemic impacts on participants and the ripple effects that quantitative data alone cannot capture. A side-by-side joint display of the quantitative and qualitative findings is represented in Table 4.

**Table 4:** Joint display: experiences of police interactions related to pandemic restrictions

Quantitative studies	Qualitative studies (*n* = 89)
**Police interactions (125/1**, **130)** 125 participants (11%) described interactions with police in relation to pandemic restrictions.	Thirty-eight participants (44%) described pandemic-related interactions with police. Participants felt an increased presence of police in drug market areas and low socio-economic suburbs where they lived. Many participants felt targeted, harassed, and unfairly treated by police due to criminal legal histories and/or they used drugs.
**Reasons for police interactions** Gatherings in homes: 10% (*n* = 12) Gatherings in public places: 9% (*n* = 11) Failing to wear facemask: 17% (*n* = 21) Breaching curfews: 26% (*n* = 33) Breaching 5 km radius*: 13% (*n* = 16) Leaving home - non-essential purposes*: 7% (*n* = 9) Other: 13% (*n* = 17)	No participants described interactions with police in relation to gatherings in homes or public places. Seven participants (18%) received A$200 fines for failing to wear a facemask, including one participant with a medical exemption. Most interactions with police were related to breaching curfews, travel restrictions, and leaving home for non-essential purposes. Six participants (16%) were fined for breaching curfews, and 18 (47%) for being outside restricted travel zones or leaving home for non-essential purposes.
**Outcomes of police interactions** Simple discussion: 41% (*n* = 51) Given a warning: 21% (*n* = 28) Issued with a fine: 30% (*n* = 38) Arrested: 15% (*n* = 19)	Several participants believed they were unnecessarily stopped and searched by police; most described the experience as familiar and non-surprising. Seven participants (18%) were given advice or warnings by police about following public health orders. 31 participants (35%) received at least one COVID-19 fine (most were A$1,652); none had the financial means to pay fines. Four participants applied to have fines waived; only one was successful but was required to complete compulsory counselling. One participant completed a prison sentence (in parallel with an existing sentence) in lieu of paying a fine. Three participants received court orders for unpaid fines. Nine (23%) participants were arrested, including four for allegedly breaching public health orders; five arrests were for offences related to social isolation and/or struggling financially and four for previous charges when approached about pandemic restrictions.

*Free text survey responses

## Discussion

Our study extends understandings about the adverse impacts of COVID-19 pandemic policing on disadvantaged and structurally marginalized populations. Our findings match those of other studies that point to how existing public order policing towards the ‘usual suspects’ was intensified during the pandemic (Boon-Kuo et al., 2021a, b). In doing so, we have addressed a gap in the literature for a population which to date has received little empirical attention. Findings illustrate how law enforcement measures introduced to protect the health and well-being of the public, served to entrench and widen existing structural inequalities for people who use drugs (Chandler et al., 2009).

Accurate data on the numbers of people who received COVID-19 fines in the general community in Victoria is not publicly available for comparison to our study findings. However, based on crude estimates, findings suggest our study participants were fined at a rate almost three times (38/1,130; 2.9%) higher than that of the general community (1%) given approximately 50,000 COVID-19 fines were administered in the state of Victoria (Taylor, 2022) in a population of approximately 5 million adults (Australian Bureau of Statistics, 2021). Furthermore, our findings support existing evidence, that people who experienced interactions with police during the pandemic were more likely to experience socio-demographic disadvantage.

As highlighted above, people who use drugs are more susceptible to heightened police scrutiny, surveillance, interference and harassment, because of their visibility in areas where drugs are often purchased and used (Dixon & Maher, 2005; Gaston et al., 2023; Stevens et al., 2015). Our findings highlight how previous participant experiences such as these, created fear that they would be targeted for breaching pandemic rules. Furthermore, findings illustrate how law enforcement measures used during the pandemic, did, in effect, produce and exacerbate these very outcomes. We have illustrated how the increased discretionary power police were afforded during the pandemic provided an opportunity for the amplification of differential and discriminatory treatment towards people who use drugs. Accounts of experiencing targeted and unfair treatment, including harassment, non-use of discretion in instances where COVID-19 fines may have been avoided, the over-use of stop and search powers (often under the presumption that drugs would be detected), and disregard for honest disclosures, highlight how pandemic-related policing helped perpetuate and exacerbate discriminatory and selective policing practices against people who use drugs. Experiences of being punished for attempting to exercise their right to healthcare—i.e., being fined for travelling to collect methadone or sterile injecting equipment when they possessed supporting medical exemptions—is further evidence of the harmful compounding criminalizing effects on participants health and social well-being, including exacerbating mistrust, and potentially increasing their exposure to infectious disease transmission, injecting related injuries and disease, and overdose (Alang et al., 2021; Maher & Dixon, 2017). Participants’ belief that the pandemic provided an “excuse” for police to increase surveillance and to treat people who use drugs unfairly, is also evidence of the stigmatising effects of these practices and the mis-guided use of emergency powers to over-ride individual health needs (Boon-Kuo et al., 2021a, b; Hopkins & Popovic, 2023).

Our study not only highlights the disproportionate impacts of pandemic policing on people who use drugs but also demonstrates how the criminalization of drug use during the pandemic compounded existing structural inequalities and increased vulnerability for an already marginalized population. We advocate for the decriminalization of drug use—the removal of criminal penalties for drug possession and use—as an approach for addressing these harms (Hughes et al., 2019). Decriminalization has the potential to address some of the inequalities highlighted in our study, ensure fairer treatment, and align public health measures with the goal of supporting, rather than penalizing, people who use drugs. Framing drug use as a public health and a human rights issue rather than a criminal matter could help curb police overreach, reduce economic harm from punitive fines, and foster greater trust in public health initiatives over the long term (Hughes et al., 2019; Lewis et al., 2022; Seear, 2003; Stevens et al., 2024).

Participant narratives highlight how COVID-19 fines were used in situations that could potentially have been addressed by a warning or formal caution (Mabin, 2023), with little obvious consideration given to the circumstances or vulnerabilities of individual needs. It is widely acknowledged that penalty notices have particular implications and punitive net-widening effects that disproportionately impact marginalized and disadvantaged communities (Brown et al., 2017; Mabin, 2023; Quilter & Hogg, 2018); our findings support this argument. We have drawn attention to how the imposition of fines during the pandemic was disproportionate, aggravated income inequalities, and placed an additional economic burden on an already financially stressed population. Unpaid fines for even minor infringements can lead to ongoing adverse impacts, including entering a vicious cycle of debt by accruing interest on top of original fines; an order to appear before a magistrate or judge or to undertake community work; and/or as a last resort, being sentenced to prison (Victoria Legal Aid, 2023); some impacts which are evidenced in our findings. The fixed nature of penalty notices, which is not proportionate to people’s incomes, means that economically vulnerable individuals are punished more harshly, without adjustments being made to account for the amount imposed. Furthermore, the financial value of COVID-19 fines (e.g., A$1,652 for breaching stay-at-home orders; A$4,957 for ‘unlawful gatherings’; and A$10,000 for ‘repeat offenders’ [Victoria Legal Aid, 2023]) far exceeded that of other commonly issued fines in Victoria (e.g., A$481for driving through a red traffic light; A$240 for exceeding the speed limit by < 10 km/hr; and A$577 for using a mobile phone while driving [Victorian Government, 2024]). Although recipients of COVID-19 fines were technically afforded opportunities to have their fines reduced, to pay by instalments, or to have them withdrawn or challenged in court if deemed wrong or unfair (Victoria Legal Aid, 2023), participant narratives highlight how structural disincentives made accessing the court process untenable (Mabin, 2023). That is, people on low incomes, like those in our study, often lack the social and/or financial capital to contest fines, including lacking access to computer technology or mobile phone credit, or knowledge and confidence to complete application processes. Moreover, as demonstrated in participant narratives, past discriminatory experiences with the criminal legal system meant they had little confidence in the process of appeal, which increased their fear of escalating criminalizing effects and decreased their motivation to challenge the fines they were issued.

Our study demonstrates how public health emergency responses centred around policing and criminalization exacerbated harms for people who use drugs, with detrimental effects enduring beyond the pandemic. Findings provide useful learnings for future pandemics and public health emergencies, to ensure the health and well-being of criminalized populations, such as people who use drugs, is protected. We argue, as others have (Leal et al., 2023; Mabin, 2023), that law enforcement, in such situations, should not be the default model for achieving public health compliance—particularly for people who are already at the ‘sharp end’ of criminalizing effects— and that the harms participants experienced could have been avoided or mitigated by police agencies. We recommend that future responses to pandemics and public health emergencies should involve the meaningful partnering of police with drug user peer organisations and harm reduction services to identify and develop alternative non-coercive community-based approaches. For example, policies and programs that prioritize harm reduction and ensure public health measures are enforced with sensitivity to social inequalities, such as peer-led safety, health and wellbeing patrols, are more likely to encourage and support public health order compliance than punitive measures (Laufs & Waseem, 2020), and at the same time to potentially reduce fear, stigma and unnecessary criminalization. Furthermore, participant narratives demonstrate how assumptions by police about people who use drugs led to discriminatory treatment. Explicitly addressing the stigmatising attitudes and assumptions about people who use drugs through policing reforms and training may help to reduce the harms of criminalization, including promoting the use of discretionary powers in ways that are trauma-informed and that consider how drug use, and individual circumstances such as low socio-economic status and poor health interact to increase vulnerability (Beckett et al., 2022; Birch, 2024).

Furthermore, to address the ongoing financial and emotional hardship and criminalizing burden arising from the inequitable distribution of COVID-19 fines among marginalized populations, we recommend that all Australian governments follow the steps of the NSW Government and waive all COVID-19 fines (Hopkins & Popovic, 2023, NSW Government, 2024). In the absence of the above response, we recommend that courts consider questions of equity and dismiss any unjust attempts at enforcing unpaid penalties related to COVID-19 fines (Brooks & Lopez, 2020).

### Study limitations and strengths

Our study has some limitations. Findings are not generalizable to the population of people who use drugs, within or outside Australia, because Melbourne and Victoria experienced more stringent public health responses and longer lock-down periods than most other locations during the pandemic, which may have resulted in higher rates of police interactions for our sample. Furthermore, although we have argued that the participants in our study were over-policed during the pandemic, we understand that without accurate comparative data with the general community or comparative data about participants interactions with the police prior to the pandemic, we cannot definitively state this was the case. On the other hand, SuperMIX and VMAX surveys did not provide opportunities for participants to indicate how many times they had had interactions with police or to report more than one type of interaction, which means the number of participants who reported receiving a fine may be an underestimate. Finally, we were unable to contact all participants who reported receiving COVID-19 fines, thus our final sample who was contacted may be biased.

Despite these limitations, our study has several strengths. The novel ethno-epidemiological approach we used (Walker et al., 2024). allowed us to recruit a more representative sample into the qualitative study than would have been possible using purposive or snowball sampling, and thus improved the rigour and trustworthiness of the qualitative evidence gathered. The comprehensive mixed methods approach we used (Fetters et al., 2013; Guetterman et al., 2015) allowed us to capitalize on the strengths of both quantitative and qualitative methods. Furthermore, being able to compare quantitative and qualitative findings with each other through our integrative design, helped create a more holistic and comprehensive understanding of the issues under examination. Finally, as the first published study, to our knowledge, to examine the impacts of pandemic policing on people who use drugs, our study addresses an important gap in the literature.

## Conclusions

Research findings extend understandings of the ways pandemic policing created opportunities for the intensification of discriminatory and criminalizing effects on members of already marginalized and stigmatized groups. We have demonstrated how for many participants in our study, law enforcement measures introduced during the pandemic—including stop and search practices and the issuing of COVID-19 fines—were used in ways that aggravated existing social, economic and health inequities and harms. Our findings provide insights for future health emergencies and the need to ensure public health responses are designed in ways that protect rather than exacerbate the health and safety of marginalized groups, including people who use drugs.

## Data Availability

The data that support the findings of this study are available from the authors upon reasonable request .
